# Group cohesion in online and in-person psychotherapy in a randomized control trial for cancer survivors

**DOI:** 10.1016/j.ijchp.2025.100635

**Published:** 2025-10-15

**Authors:** Alejandra Arizu-Onassis, Joan C. Medina, María Lleras de Frutos, Aida Flix-Valle, Maria Serra-Blasco, Laura Ciria-Suarez, Cristian Ochoa-Arnedo

**Affiliations:** a Psycho-oncology and Digital Health Group, Institut d´Investigació Biomédica de Bellvitge, Av. Gran Via de L’Hospitalet 199-203, L’Hospitalet del Llobregat, Spain; b Department of Clinical Psychology and Psychobiology, Universitat de Barcelona, Pg. de la Vall d'Hebron 171, Horta-Guinardó, Barcelona, Spain; c ICOnnecta’t Digital Health Program, Institut Català d´Oncologia, Av. Gran Via de L’Hospitalet 199-203, L’Hospitalet del Llobregat, Spain; d Department of Psychology and Education Sciences, Universitat Oberta de Catalunya, Rambla del Poblenou 154-156, Sant Martí, 08018 Barcelona, Spain; e Psycho-Oncology Department, Institut Català d´Oncologia, Av. Gran Via de L’Hospitalet 199-203, L’Hospitalet del Llobregat, Spain; f CIBERSAM, Instituto de Salud Carlos II, Av. Monforte de Lemos 3-5, Pabellón 11, Planta 0, Madrid, 28029, Spain

**Keywords:** Psycho-oncology, Online group psychotherapy, Group cohesion, Positive psychotherapy, Randomized controlled trial, Psico-oncología, Psicoterapia grupal en línea, Cohesión grupal, Psicoterapia positiva, Ensayo controlado aleatorio

## Abstract

**Objective:**

To explore the role of group cohesion (GC) in-person positive group psychotherapy (PPPC) and online positive group psychotherapy (OPPC).

**Method:**

A sample of 126 female cancer survivors experiencing emotional distress following primary oncological treatment was randomized to PPPC or OPPC. Measures of emotional distress, post-traumatic stress, and post-traumatic growth were taken at pre-treatment, post-treatment (after 12 weeks), and 3-month follow-up (from post-treatment), with GC assessed at post-treatment for this secondary analysis.

**Results:**

There were no significant differences in GC levels between the PPPC and OPPC groups. Higher GC was associated with greater reductions in depressive symptoms (*b=* -0.80, CI(95 %)= -1.18 – -0.42, *p*< 0.001) and post-traumatic stress (*b=* -1.38, CI(95 %)= -2.42 – -0.34, *p*< 0.010) in both modalities. Participants with higher GC reported immediate post-traumatic growth at post-treatment, while those with lower GC achieved similar growth levels by 3-month follow-up. No specific sociodemographic or clinical variables were associated with higher GC.

**Conclusions:**

In group psychotherapy for cancer survivors, GC is associated with a more pronounced reduction of depressive symptoms and post-traumatic stress, and with earlier post-traumatic growth in both OPPC and PPPC. Findings suggest that all cancer survivors have equal potential to develop GC, regardless of clinical or sociodemographic characteristics.

## Introduction

Cancer is a common disease with a significant impact on patients’ lives. It has multiple consequences, such as substantial functional and cognitive impairment or difficulty returning to daily life (Yen et al., 2024). Up to 50 % of cancer patients also experience high levels of emotional distress, primarily anxiety and depression, during their illness (Naser et al., 2021). The post-treatment phase is particularly stressful due to the loss of frequent medical contact and the cessation of active treatment. High emotional distress in oncology patients is linked to greater physical symptoms and functional impairment (Grassi et al., 2023). In addition, it leads to decreased quality of life (Aitken & Hossan, 2022; Dionisi-Vici et al., 2021; Guarino et al., 2020), lower adherence to treatment (Khalili et al., 2021), less self-care and healthy lifestyle, and reduced overall survival (Abdelhadi, 2023; Clark et al., 2021).

Focusing on the post-treatment stage, early access to psycho-oncology treatment has shown to enhance psychological adaptation and quality of life (Guarino et al., 2020; Paley et al., 2023). Indeed, a recent study by Ochoa-Arnedo et al. (2022) showed that a one-point decrease on the Hospital Anxiety and Depression Scale (Zigmond & Snaith, 1983) following psychotherapy predicted a lower risk of 5-year recurrence. Hence, the provision of psychological support throughout the cancer journey, including post-treatment and extended survivorship, is crucial (Khajoei et al., 2023).

Recently, the focus in treating cancer survivors has shifted to promoting post-traumatic growth (PTG) due to its predictive role in reducing depression, anxiety, and post-traumatic stress symptoms (Vrontaras et al., 2023). Cognitive-behavioral therapy has traditionally been the primary therapeutic approach for individuals with medical conditions (Guarino et al., 2020; Paley et al., 2023). However, positive psychotherapy for cancer survivors (PPC), which aims to enhance personal resources, positive emotions, and the construction of personal and spiritual meaning even after traumatic experiences (post-traumatic growth), has emerged as an equally effective approach, particularly suited to the survivorship period (Lleras de Frutos et al., 2020).

In terms of treatment modality, group psychotherapy has become popular for the psychosocial treatment of cancer patients due to its advantages over individual psychotherapy. It facilitates therapeutic factors such as social support, vicarious learning, and constructive compassion, which are linked to better psychosocial outcomes (Malat et al., 2024). In addition to providing economic benefits, evidence shows that it can lead to systematic improvements in fatigue, pain, anxiety, quality of life, coping skills, adjustment (Wei et al., 2016), cortisol levels, and depression (Rosendahl, 2021), as well as enhancing the value patients place on their own lives (Lleras de Frutos et al., 2020).

In any group psychotherapy, group cohesion (GC) is known to be one of the key therapeutic processes and it has been associated with better outcomes (Christensen et al., 2021. GC is characterized by strong bonds, a sense of belonging, attraction to the group and its members, and goal coordination (American Psychological Association, n.d., Definition 1). High GC increases adherence and contributes to therapeutic outcomes such as symptom reduction and improved interpersonal functioning, regardless of the intervention type (Burlingame et al., 2018). It is related to therapeutic outcomes to the same extent as therapeutic alliance in individual therapy (*r* = 0.28) (Rosendahl et al., 2021). Empirical studies of GC in cancer psychotherapy began with Andersen et al. (2007), who found a strong link between GC and therapy outcomes. Higher GC was associated with significant improvements in emotional distress, physical activity, and performance over time, with effects lasting up to 12 months after psychotherapy.

However, to date, most group therapies have been conducted in-person, which poses some limitations for oncology patients who often face physical discomfort, socioeconomic challenges, and reluctance to visit healthcare facilities frequently. These barriers can limit access to consistent care, increase costs, and reduce convenience. To address these challenges, information and communication technologies have been integrated into the healthcare system over recent decades. Video-consultation platforms, mobile apps, web-based programs, and wearable devices now enable remote support and monitoring, enhancing accessibility and reducing healthcare costs (Caminiti et al., 2023). Initially, online support for cancer patients emerged as written, asynchronous support groups (Finfgeld, 2000). Over time, high-quality group psycho-oncology treatments have been conducted via videoconferencing, proving to be a useful and effective alternative to in-person interventions (Lleras de Frutos et al., 2020).

Despite the clear link between GC and positive psychotherapy outcomes, research analyzing GC in online group psychotherapy for cancer patients is scarce (McGill et al., 2017). There is also a lack of clarity regarding the variables (treatment modality, group composition, patient sociodemographic and clinical characteristics) that may facilitate the development of this GC (Malat et al., 2024). Understanding how GC influences therapeutic outcomes in this context and identifying the variables that promote it is vital for optimizing the efficacy of online group psychotherapy for cancer patients. Therefore, further research is needed to fill this gap and provide a deeper understanding of the dynamics at play.

Altogether, this study aims to compare levels of GC between patients who participated in-person group psychotherapy and those who participated in online group psychotherapy; and to analyze whether GC influences the efficacy of online and/or in-person PPC on emotional distress, post-traumatic stress, and post-traumatic growth. Finally, a complementary objective is to explore which clinical and sociodemographic variables predict the development of greater GC.

## Methods

### Design

This is a secondary, modified intention to treat (mITT) analysis of a randomized clinical trial (NCT03010371) evaluating the efficacy of online PPC compared to in-person. A two-arm pragmatic RCT was conducted within the routine practice of a monographic cancer center in northeastern Spain. This design was selected to enhance internal-external validity balance. The study protocol was approved by the institutional ethics committee (PR104/13) and all patients signed an informed consent prior to enrollment. Participants in the experimental group received online positive group psychotherapy for cancer (OPPC), while patients assigned to the control group received in-person positive group psychotherapy for cancer (PPPC). A full description of the study methodology is provided in Lleras de Frutos et al. (2020). The data analyzed here include a subsample of the primary trial that provided GC scores. After the study started, the protocol was amended to measure GC, as it became a variable of interest to the research group, which decided to include it in all ongoing studies. As the primary trial was an RCT, this manuscript follows CONSORT reporting guidelines (Schulz et al., 2010).

### Participants

Between January 2016 and January 2019, the primary RCT recruited women with different cancer diagnoses. They were referred to the study by medical oncologists or nurses if they experienced emotional distress after their primary cancer treatment. A psycho-oncologist conducted in-person interviews and collected data using an online questionnaire, including sociodemographic and clinical information and psychometric instruments. If participants met specific criteria (i.e., over 18 years of age, completion of primary cancer treatment, ≥10 on the Hospital Anxiety and Depression Scales (HADS) total score, internet access, and Spanish language skills), they were invited to join the study.

### Procedure

The study aimed to include as many users as possible. A computer-generated randomization table was used, and patients were invited to accept random allocation after both treatment modalities were explained.

Group therapy was led by two clinical psychologists with expertise in the PPC treatment protocol (Ochoa Arnedo & Maté Méndez, 2010), both in PPPC and OPPC.

### Instruments

Emotional distress: The Hospital Anxiety and Depression Scale (HADS) measures the presence and intensity of anxiety and depression symptoms in individuals with physical illness. It consists of 14 items, with 7 items assessing anxiety and 7 items assessing depression on a four-point Likert scale (0–3). The subscale scores range from 0 to 21, and total score ranges from 0 to 42. Higher scores indicate more severe anxiety or depression symptoms. A cut-off of ≥ 10 on HADS total scale can be used for screening purposes, and a change of ≥2 points has been used as a cut-off point to assess clinical change (Vaganian et al., 2020). It has been validated in Spanish in the oncology population (Costa-Requena et al., 2009), demonstrating good reliability (*α* = .82 and *α* = .84 for the anxiety and depression subscales, respectively).
Post-traumatic stress: The Post-Traumatic Stress Disorder Checklist – Civilian Version (PCL-C) is a 17-item self-report questionnaire designed to assess post-traumatic stress disorder. Each item is scored from 1 to 5. Total score ranges from 17 to 85, with higher scores indicating greater post-traumatic stress symptoms. A recommended cut-off score of ≥ 44 is used to detect clinical cases**.** The validated Spanish version (Costa-Requena & Gil Moncayo, 2010) shows good reliability (*α* = .90).
Post-traumatic growth: The Post-Traumatic Growth Inventory (PTGI) is a 21-item self-report questionnaire designed to assess positive changes experienced after trauma. Each item is scored from 0 to 5. Hence, total scores ranges from 0 to 105, where higher scores represent more significant post-traumatic growth. Its validation in Spanish among oncology patients showed excellent reliability (*α* = .95) (Costa-Requena & Gil Moncayo, 2007).
Group cohesion: Patient perceptions of GC were assessed using two items following Andersen (2007). Though this instrument has not yet been validated, it was selected for this study because it had been previously used in cancer patients, and it focuses on key aspects of group engagement. Items included the following questions: “How involved did you feel in this group experience?” and “How supported did you feel by this group?”. Each item was rated on an 11-point Likert scale ranging from 0 (not at all) to 10 (extremely). A participant’s overall GC score was calculated as the average score across these items.

Measures of emotional distress, post-traumatic stress and growth were taken at pre-treatment, post-treatment (after 12 weeks), and 3-month follow-up (from post-treatment); while GC was assessed once at post-treatment.

### Intervention

PPPC is a therapist-led group intervention aimed at reducing emotional distress and fostering post-traumatic growth (PTG) in cancer survivors. It consists of 12 weekly in-person sessions (90–120 min each) for groups of 8–12 participants, organized into four modules targeting emotional regulation, coping, and facilitating positive life changes.

OPPC offers the same content via secure videoconferencing (ViTAM®), with 11 weekly virtual sessions and one final in-person session. Due to platform constraints, OPPC groups are smaller (5–6 participants). Secure, real-time interaction is supported, with technical assistance and encrypted data to ensure confidentiality.

### Data analyses

For descriptive analyses, categorical variables were displayed as numbers of cases and corresponding percentages. Continuous variables were reported as means and standard deviations (SDs) or medians and interquartile ranges, depending on their distribution.

Multiple imputation by chained equations was used to account for missing data in the GC, HADS, PCL-C, and PTGI measures (Sterne et al., 2009). The assumption that the missing values were missing at random was considered suitable because no patterns were found among the missing values (Jolani et al., 2015). Fifty iterations of imputation were performed.

Kruskal-Wallis testing was used to compare GC at post-treatment between the OPPC and PPPC groups. To analyze the effect of GC on the evolution of emotional distress (HADS total score and subscales), post-traumatic stress, and post-traumatic growth between post-treatment and 3-month follow-up, linear mixed-effects models (LMMs) were calculated. For every outcome measure, patients were added as random effects, and time, treatment modality, GC, and the interaction between these three variables were added as fixed effects. LMMs were adjusted for age, education, and work status, replicating the procedure used in the original RCT analyses to assess treatment efficacy.

Finally, to explore potential variables associated with GC (age, educational level, civil status, job status, disease stage, and type of primary oncological treatment), independent univariate linear regression models were estimated for each variable of interest. The statistical software R, version 4.3.3 for Windows, was used for data processing and analysis (R Core Team, 2017).

## Results

**Fig. 1:**
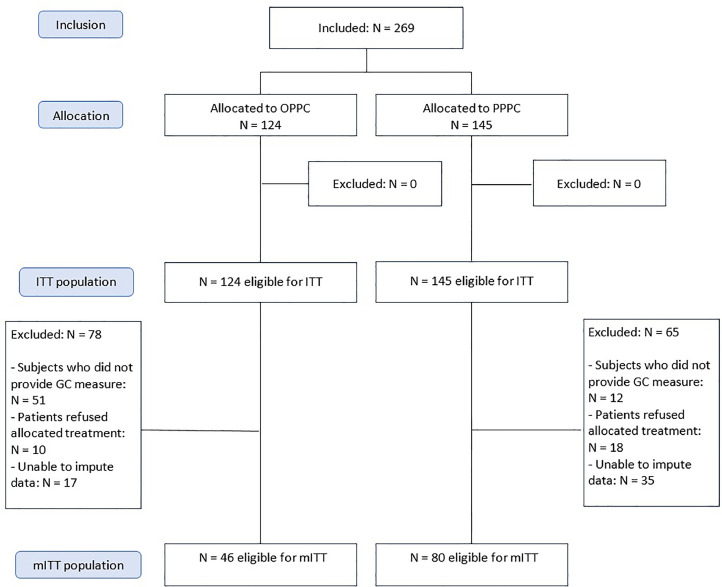
CONSORT Flowchart for eligibility and inclusion in this study (mITT population).

Note: OPPC = Online positive group psychotherapy for cancer; PPPC = in-person positive group psychotherapy for cancer; ITT = intention to treat; GC = group cohesion; mITT = modified intention to treat.

A total of 269 women were included in the main RCT, of whom 124 were allocated to OPPC and 145 to PPPC. 78 OPPC participants and 65 PPPC participants were excluded from this secondary analysis for the following reasons: inability to impute missing data (17 in OPPC; 35 in PPPC), refusal to accept the allocated treatment (10 in OPPC; 18 in PPPC), and failure to complete the GC measure (51 in OPPC; 12 in PPPC). Thus, a final sample of 126 participants was analyzed: 46 participants allocated to OPPC (13 groups) and 80 participants allocated to PPPC (13 groups). Details of the participant flowchart are provided in Fig. 1. The same number of OPPC and PPPC groups were included in this secondary analysis. However, because OPPC groups had less participants (5–6 in OPPC compared to 8–12 in PPPC), due to the online platform limitations, samples differ in size. Only women were included in the main RCT to use the PPC protocol in its validated population and to guarantee group homogeneity (Lleras de Frutos et al., 2020).

Sociodemographic and clinical variables of the included (*N* = 126) and excluded (*N* = 143) participants are shown in Table 1. No clear differences have been found.

**Table 1:** Sociodemographic and clinical characteristics of excluded and included participants.

	Excluded	Included	N
	*N=143*	*N=126*	
**Age Mean (SD)**	50.6 (8.87)	49.2 (8.14)	269
**Civil Status, N (%):**			267
Single	8 (5.67 %)	8 (6.35 %)	
Divorced	30 (21.3 %)	14 (11.1 %)	
Married	93 (66.0 %)	98 (77.8 %)	
Widow	10 (7.09 %)	6 (4.76 %)	
**Education level, N (%):**			256
Basic	47 (35.6 %)	31 (25.0 %)	
Advanced	34 (25.8 %)	35 (28.2 %)	
Intermediate	51 (38.6 %)	58 (46.8 %)	
**Job status, N (%):**			252
Pasive	15 (11.7 %)	9 (7.26 %)	
Pensioner	20 (15.6 %)	26 (21.0 %)	
Unemployment benefit	18 (14.1 %)	7 (5.65 %)	
Active	20 (15.6 %)	15 (12.1 %)	
Leave	55 (43.0 %)	67 (54.0 %)	
**Months (mean) since diagnosis (SD)**	22.55 (15.68)	19.81 (16.13)	262
**Stage, N (%):**			259
Stage 0	8 (5.80 %)	5 (4.13 %)	
Stage I	45 (32.6 %)	48 (39.7 %)	
Stage II	55 (39.9 %)	39 (32.2 %)	
Stage III	21 (15.2 %)	24 (19.8 %)	
Stage IV	9 (6.52 %)	3 (2.48 %)	
Disease not measured in stages	0 (0.00 %)	2 (1.65 %)	
**Preference, N (%):**			269
Accepted randomization	99 (69.2 %)	126 (100 %)	
Preferred online	16 (11.2 %)	0 (0.00 %)	
Preferred face-to-face	28 (19.6 %)	0 (0.00 %)	
Allocated group, N ( %)	129 (91.5 %)	114 (91.2 %)	266
**Primary oncological treatment**			
Surgery, N ( %)	107 (75.9 %)	98 (77.8 %)	267
Radiotherapy, N ( %)	103 (73.0 %)	91 (72.2 %)	267
Chemotherapy, N ( %)	81 (57.9 %)	75 (59.5 %)	266
Hormone Therapy, N ( %)	3 (2.14 %)	3 (2.38 %)	266
Bone Marrow Transplant, N ( %)	16 (11.4 %)	12 (9.52 %)	266
Brachytherapy, N ( %):			259

Note: SD = standard deviation.

Additionally, the sociodemographic and clinical characteristics of participants allocated to PPPC and OPPC are summarized in Table 2. Participants in the OPPC and PPPC groups exhibited only modest sociodemographic differences. The mean age was slightly higher in the PPPC group (50.9 years) compared to the OPPC group (46.3 years). In both groups, most participants were married (PPPC: 80.0 %; OPPC: 73.9 %), with smaller proportions being divorced, single, or widowed. The PPPC group had a higher proportion of individuals with basic education (30.8 %), whereas the OPPC group had a greater proportion with advanced education (39.1 %).

**Table 2:** Sociodemographic and clinical characteristics of participants in both treatment modalities.

	PPPC	OPPC	N
	*N=80*	*N=46*	
**Age Mean (SD)**	50.9 (7.89)	46.3 (7.83)	126
**Civil Status, N (%):**			126
Single	4 (5.00 %)	4 (8.70 %)	
Divorced	8 (10.0 %)	6 (13.0 %)	
Married	64 (80.0 %)	34 (73.9 %)	
Widow	4 (5.00 %)	2 (4.35 %)	
**Education level, N (%):**			124
Basic	24 (30.8 %)	7 (15.2 %)	
Advanced	17 (21.8 %)	18 (39.1 %)	
Intermediate	37 (47.4 %)	21 (45.7 %)	
**Job status, N (%):**			124
Pasive	8 (10.3 %)	1 (2.17 %)	
Pensioner	15 (19.2 %)	11 (23.9 %)	
Unemployment benefit	3 (3.85 %)	4 (8.70 %)	
Active	8 (10.3 %)	7 (15.2 %)	
Leave	44 (56.4 %)	23 (50.0 %)	
**Months (mean) since diagnosis (SD)**	19.88 (16.30)	20.70 (16.80)	124
**Stage, N (%):**			121
Stage 0	5 (6.41 %)	0 (0.00 %)	
Stage I	31 (39.7 %)	17 (39.5 %)	
Stage II	24 (30.8 %)	15 (34.9 %)	
Stage III	15 (19.2 %)	9 (20.9 %)	
Stage IV	2 (2.56 %)	1 (2.33 %)	
Disease not measured in stages	1 (1.28 %)	1 (2.33 %)	
**Primary Oncological Treatment**			
Surgery, N ( %)	72 (91.1 %)	42 (91.3 %)	125
Radiotherapy, N ( %)	61 (76.2 %)	37 (80.4 %)	126
Chemotherapy, N ( %)	56 (70.0 %)	35 (76.1 %)	126
Hormone Therapy, N ( %)	45 (56.2 %)	30 (65.2 %)	126
Bone Marrow transplant, N ( %)	1 (1.25 %)	2 (4.35 %)	126
Brachytherapy, N ( %)	10 (12.5 %)	2 (4.35 %)	126

Note: OPPC = online positive group psychotherapy for cancer; PPPC = in-person positive group psychotherapy for cancer; SD = standard deviation.

The mean time since diagnosis was comparable between groups (PPPC: 19.88 months; OPPC: 20.70 months). Group psychotherapy for cancer survivors was offered following completion of primary oncological treatment (typically occurring approximately 12 months after diagnosis) to individuals presenting emotional distress. Owing to limited resources, initiation of therapy was generally preceded by a waiting period of around 6 – 7 months. Most participants in both groups were survivors of stage I or II cancer, approximately 91 % had undergone oncological surgery, 76–80 % had received radiotherapy, and 70–76 % had received chemotherapy. Age, educational level, as well as job status (because of statistical differences in the original RCT), were controlled for in subsequent analyses.

When comparing GC between OPPC and PPPC, analyses showed no statistically significant differences, indicating that similar levels of GC were perceived by participants in both modalities (see Table 3).

**Table 3:** Comparison of Group Cohesion between treatment modalities.

	PPPC	OPPC	*p*. Overall
**GC, Median (*Q1; Q3*)**	9.00 (7.50; 10.00)	9.50 (8.12; 10.00)	0.246

Note: GC = group cohesion; Q1 = quartile 1; Q3 = quartile 3; OPPC = online positive group psychotherapy for cancer; PPPC = in-person positive group psychotherapy for cancer.

*p < .05.

Regarding the role of GC in the evolution of emotional distress, post-traumatic stress, and post-traumatic growth scores, the results of the LMMs are detailed below in Table 4.

**Table 4:** Linear mixed models results: the influence of Group Cohesion on the evolution of outcome measures.

		Adjusted Models			
	Predictors	Estimates	std. Error	CI	*p*
**HADS total score**	Time x Treatment modality	0.82	1.38	−1.90 – 3.54	0.553
	Time x Group Cohesion	−0.45	0.38	−1.22 – 0.30	0.241
	Treatment modality x Group Cohesion	−0.10	0.61	−1.30 – 1.10	0.873

**HADS anxiety subscale**	Time x Treatment modality	0.84	0.87	−0.88 – 2.56	0.337
	Time x Group Cohesion	−0.12	0.24	−0.60 – 0.36	0.615
	Treatment modality x Group Cohesion	−0.06	0.42	−0.88 – 0.76	0.894

**HADS depression subscale**	Time x Treatment modality	0.26	0.74	−1.20 – 1.72	0.725
	Time x Group Cohesion	−0.80	0.19	−1.18 – −0.42	<0.001
	Treatment modality x Group Cohesion	0.32	0.38	−0.42 – 1.06	0.394

**PCL-C total score**	Time x Treatment modality	−2.38	1.94	−6.20 – 1.43	0.220
	Time x Group Cohesion	−1.38	0.53	−2.42 – −0.34	0.010
	Treatment modality x Group Cohesion	0.92	1.00	−1.05 – 2.90	0.359

**PTGI total score**	Time x Treatment modality	3.54	3.26	−2.88 – 9.95	0.279
	Time x Group Cohesion	−2.08	0.89	−3.84 – −0.33	0.020
	Treatment modality x Group Cohesion	0.24	1.63	−2.97 – 3.45	0.885

Note: HADS = Hospital Anxiety and Depression Scale; PCL-*C* = Post-Traumatic Stress Disorder Checklist – Civilian Version; PTGI = Post-Traumatic Growth Inventory; std. Error = standard error; CI = confidence interval.

*p*< .05.

Analyses showed that the evolution of HADS total and anxiety scores over time (between post-treatment and 3-month follow-up) did not differ significantly based on the interaction between treatment modality and time, GC and time, GC and treatment modality.

**Fig. 2:**
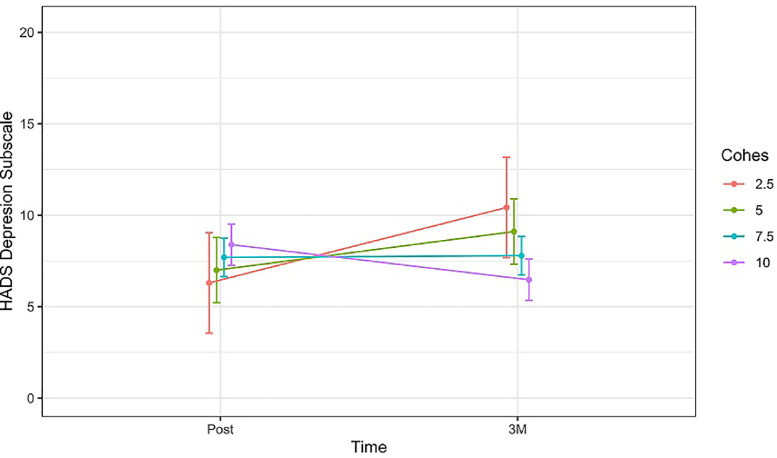
Change in the estimated mean of HADS Depression subscale [95 %CI] over time (between post-treatment and 3 months follow-up), comparing patients with different levels of GC.

Note: HADS = Hospital Anxiety and Depression Scale; CI = confidence interval; Post = post-treatment assessment; 3 *M* = 3-month follow-up

**Fig. 3:**
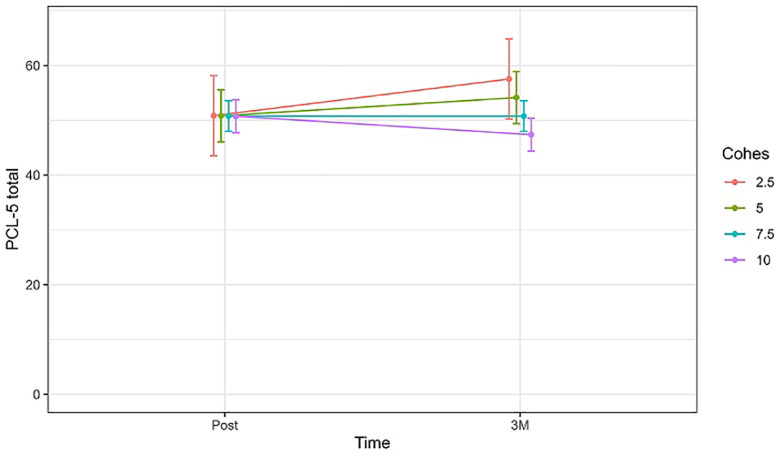
Change in the estimated mean of PCL-C total scores [95 %CI] over time (between post-treatment and 3 months follow-up), comparing patients with different levels of GC.

Note: PCL-*C* = Post-Traumatic Stress Disorder Checklist – Civilian Version; CI = confidence interval; Post = post-treatment assessment; 3 *M* = 3-month follow-up

However, the evolution of the HADS depression subscale and the PCL-C over time appeared to differ significantly based on the interaction between GC and time, showing that greater GC levels after psychotherapy were associated with more pronounced reductions in depressive (*b* = −0.80, CI(95 %) = −1.18 – −0.42, *p* < 0.001) and post-traumatic stress symptoms (*b* = −1.38, CI(95 %) = −2.42 – −0.34, *p* < 0.010) between post-treatment and 3-month follow-up (see Fig. 2, Fig. 3).

**Fig. 4:**
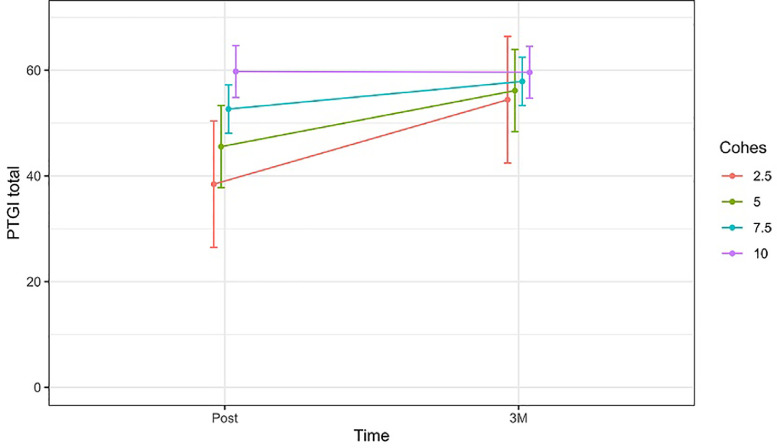
Change in the estimated mean of PTGI total scores [95 %CI] over time (between post-treatment and 3 months follow-up), comparing patients with different levels of GC.

Note: PTGI = Post-Traumatic Growth Inventory; CI = confidence interval; Post = post-treatment assessment; 3 *M* = 3-month follow-up

In the case of the PTGI, Table 4 shows that participants with lower levels of GC, presented a steeper increase of PTGI over time (*b* = −2.80, CI(95 %) = −3.84 – −0.33, *p* < 0.020). However, the interpretation of these results can be completed with Fig. 4, which shows that participants who finished psychotherapy with high levels of GC, kept them high at the 3 months follow-up. In turn, participants who experienced lower levels of GC, also reported lower PTGI scores after treatment, but these scores increased at the 3-month follow-up. This suggests that greater post-treatment GC was also associated with early development of PTG.

With respect to the influence of the sociodemographic variables adjusted for in these LMMs, only a higher level of education (advanced compared to basic) was associated with a more significant decrease in depressive symptoms (HADS depression subscale) over time (*b* = −1.96, CI(95 %) = −3.91 – −0.01, *p* = 0.049).

Regarding the influence of sociodemographic and clinical variables on GC, age is the only variable with a statistically significant effect on GC levels (*b* = −0.05; CI(95 %) = −0.08 – −0.01; *p* = 0.012), with younger age being associated with slightly higher GC. However, this estimated effect is small and clinically insignificant.

## Discussion

This exploratory study provides evidence for GC as a process variable with significant influence over therapeutic efficacy in online and in-person group psychotherapy for cancer survivors. At present, there is a paucity of research analyzing the role of GC in online psychotherapy for cancer patients. According to our results, the treatment modality (OPPC and PPPC) did not affect the levels of GC perceived by participants. This suggests not only that the online modality is equally effective (as found by Lleras et al., 2020), but also that GC, as one of the main process variables associated with therapeutic efficacy (Christensen et al., 2021; Rosendahl et al., 2021), does not seem to suffer any detriment in this treatment modality. This is consistent with the findings of McGill et al. (2017) in online support groups for adolescents and young adults with cancer.

Regarding the role of GC in therapeutic efficacy, our results show that the interaction between GC and time has a significant impact on reducing depressive symptoms and post-traumatic stress, but not anxiety symptoms and overall distress. Andersen et al. (2007) found significant positive outcomes in a similar sample of breast cancer survivors, with considerable reductions in overall distress. However, Marziliano et al. (2018) studied advanced cancer patients and found no effect of GC on depression, anxiety, or hopelessness. A relevant explanation for these discrepancies across the three studies relates to the role of the number of group therapy sessions in GC and their predictive value for distress and mental health outcomes. The Andersen et al. study involved 26 sessions, the Lleras de Frutos et al. study involved 14 sessions, and the Marziliano et al. study involved 8 sessions. The dose or number of sessions moderates GC (Burlingame et al., 2018) and mental health outcomes. Moreover, the worst prognosis in advanced cancer survivors may hinder the reduction of associated depressive and hopelessness symptoms.

In short, the improvement in both measures (depression and post-traumatic stress) in our study related to GC is clinically important, especially considering that a substantial percentage of cancer survivors continue to experience significant psychological challenges even two years after their diagnosis. Nevertheless, a more in-depth analysis of the dose (sessions) associated with GC and mental health outcomes would be necessary to elucidate how to increase and maintain positive outcomes.

In the case of PTG, the results show that participants with higher GC achieved greater PTG sooner and maintained it over time, displaying a plateau effect, while participants with lower GC did not reach these levels until the 3-month follow-up. The association between high GC and faster PTG is similar to the findings of Marziliano et al. (2018) in advanced cancer patients. This concordance highlights the relational nature of PTG, which has been analyzed in various studies (Weiss, 2004). Individuals with higher GC may achieve PTG motivated by group and relational dynamics, which favor faster and more synchronous accommodation to the oncological process. In contrast, participants with lower GC levels are also able to develop PTG, but it takes them longer. These individuals may continue to make changes in integration and accommodation to the oncological experience after therapy ends, but they may do so in a more asynchronous and probably less relational manner. Regarding the aforementioned plateau effect, this is consistent with the trajectories of PTG described by Danhauer et al. (2015), where some trajectories show stable levels of PTG over time and others rise and then plateau.

In addition, individuals with higher levels of education experienced more pronounced reductions in depressive symptoms following group psychotherapy. This finding is consistent with some studies (Alagizy, 2020; Naseri & Taleghani, 2018), while other research suggests that lower education levels are linked to higher anxiety, but not necessarily to increased depressive symptoms (Civilotti, 2021). Given these mixed results, more research is needed to clarify this issue.

Finally, younger age appears to be associated with higher GC, but only slightly; therefore, this effect is unlikely to be clinically significant. However, a possible explanation for this association could be that cancer is perceived as an unnatural experience in young people, leading to a greater potential for trauma (Aitken & Hossan, 2022) and feeling misunderstood by healthy peers. In such cases, group therapy may facilitate an increased sense of belonging, which in turn promotes GC. Nevertheless, the fact that almost no sociodemographic or clinical variables were associated with higher GC scores is consistent with previous research (Marziliano et al., 2018) and may be a promising finding. It suggests that all cancer survivors may have the same capacity to develop and experience GC when participating in online or in-person group psychotherapy. Therefore, except for special situations, cancer survivors who continue to experience emotional distress after completing oncological treatments may benefit from group psychotherapy and the GC developed during it.

## Limitations

There are several limitations to this study that should be considered when interpreting its results. First, the GC measure used has not yet been validated. However, it was selected for this study due to its similarity with other measures used in the RCT (i.e., Likert scale format), its brevity, and its previous use in studies involving cancer patients (Andersen et al., 2007).

Secondly, the sample size of the main study was estimated according to the main objective of measuring the efficacy of OPPC. Since this study is a secondary analysis, and considering that only part of the original sample completed the GC questionnaire, our results may be underpowered and should be interpreted with caution. Also, although the same number of groups were included for each treatment modality, there is an important difference between the sample size of OPPC and PPPC because OPPC groups were smaller. Moreover, other reasons for exclusion led us to remove additional participants from the analysis, resulting in differences in the allocation groups. Despite this difference in sample size, no significant differences were found in the levels of GC developed throughout OPPC and PPPC interventions. This suggests that group size did not influence GC in our study. Nonetheless, more research is needed to analyze how group size and group composition may influence the development of GC.

Finally, sociodemographic and clinical characteristics of both groups were analyzed and compared in this sub-study and no clear differences were found. Indeed, even though this study includes participants with different oncological diagnoses, it only includes female patients in the extended survival stage, which favors homogeneity. At the same time, conclusions should be limited to this population and not extended to the general oncological population (e.g., other genders or phases in the oncological process).

## Clinical implications

This study offers exploratory results that enrich our understanding of the role of GC in both online and in-person group psychotherapy for cancer survivors. Our findings advocate for the equal potential of both treatment modalities to foster GC among participants, thereby significantly enhancing therapeutic efficacy in alleviating depressive symptoms and post-traumatic stress. The study also shows that strong GC is aligned with patients experiencing post-traumatic growth earlier, synchronously with the therapeutic process. Finally, no individual sociodemographic or clinical characteristics are associated with the likelihood of developing greater or lesser GC, although age received some statistical support. Consequently, it is crucial to use interventions that promote GC to enhance therapeutic outcomes and reduce the emotional distress caused by cancer. This can be well achieved through both online and in-person group psychotherapy.

**DATA AVAILABILITY:** The data are available on request from the corresponding authors. They are not publicly available due to the privacy of the research participants.

## Author contribution

AA-O: conceptualization; methodology; data curation; analysis and results interpretation; writing of original draft. JCM: conceptualization; methodology; formal analysis; oversight of original draft. MLl-F: funding acquisition; project management; implementation of the experiment; data curation. AF-V: oversight of original draft. MS-B: oversight of original draft. LC-S: oversight of original draft. CO-A: leadership; funding acquisition; supervision; oversight of original draft. All authors: draft review and editing; approval of final version of manuscript.

## Funding information

This study was supported by the 10.13039/501100004587Carlos III Health Institute under FIS grants no PI15/01278, no PI19/01880 and PI22/01255 (co-financed by the 10.13039/501100008530European Regional Development Fund [ERDF] under the “A way to build Europe” initiative) and the 10.13039/100010686European Institute of Innovation and Technology (EIT Health) (ONCOMMUNITIES: Online Cancer Support Communities), grant/award no 20,536. Additionally, the research team received financial backing from the Emergent Agència d’Ajuts Universitaris i de Recerca of the Government of Catalonia (AGAUR), research group: Psycho-oncology and Digital Health (no. 2021 SGR 01003).

## Declaration of competing interest

The authors declare the following financial interests/personal relationships which may be considered as potential competing interests: Cristian Ochoa-Arnedo reports financial support was provided by Carlos III Health Institute. Cristian Ochoa-Arnedo reports financial support was provided by Government of Catalonia Agency for Administration of University and Research Grants. Cristian Ochoa-Arnedo reports financial support was provided by European Institute of Innovation & Technology. If there are other authors, they declare that they have no known competing financial interests or personal relationships that could have appeared to influence the work reported in this paper.
